# Supplementary material for: Photosynthesis, Anatomy, and Metabolism as a Tool for Assessing Physiological Modulation in Five Native Species of the Brazilian Atlantic Forest
Source: Plants (Basel). 2024 Jul 10;13(14):1906. doi: 10.3390/plants13141906 (PMC11280366; doi:10.3390/plants13141906)
Supplement: Supplementary file 1 [file plants-13-01906-s001.zip › Supplementary Table S1.pdf]

Supplementary Table S1. Over-representation analysis of the principal component (PC) variable loadings of metabolites with a main impact on the variance of the data set. The physiological, morphological, and metabolic parameters contributions in each PC measured in five species: *Paubrasilia echinata, Chorisia glaziovii, Clusia nemorosa, Licania tomentosa,* and *Schinus terebinthifolia* plants grows in natural environment in 2014/June to 2015/Feb in accord of a historical rainfall gradient, in Paulista, Pernambuco, Brazil.

Variable	PC1	PC2
Mannose	0.108	-0.418
Dehydroascorbate	-0.100	-0.397
Histidine	-0.094	-0.393
Asparagine	-0.072	-0.385
PP	0.103	0.353
Leucine	0.198	-0.238
Net photosynthesis	-0.149	-0.250
SLA	-0.177	-0.179
2-Oxoglutarate	-0.218	0.131
Erythritol	0.224	-0.122
Tryptophan	0.208	-0.128
3-phospho glycerate	-0.164	0.145
Fructose	0.225	-0.075
Spermidine	0.212	0.078
Malate	0.236	-0.039
myo-Inositol	0.237	-0.031
Glycerate	0.228	0.037
Shikimic acid	0.237	-0.028
Glucose-6-phosphate	0.238	-0.022
Glutarate	0.234	0.025
Tyrosine	0.238	-0.013
Palmitate	0.239	-0.011
Serine	-0.231	-0.017
Citrate	0.238	0.001
Raffinose	0.206	0.026
