# Supplementary figures and images for: Photosynthesis, Anatomy, and Metabolism as a Tool for Assessing Physiological Modulation in Five Native Species of the Brazilian Atlantic Forest
Source: Plants (Basel). 2024 Jul 10;13(14):1906. doi: 10.3390/plants13141906 (PMC11280366; doi:10.3390/plants13141906)

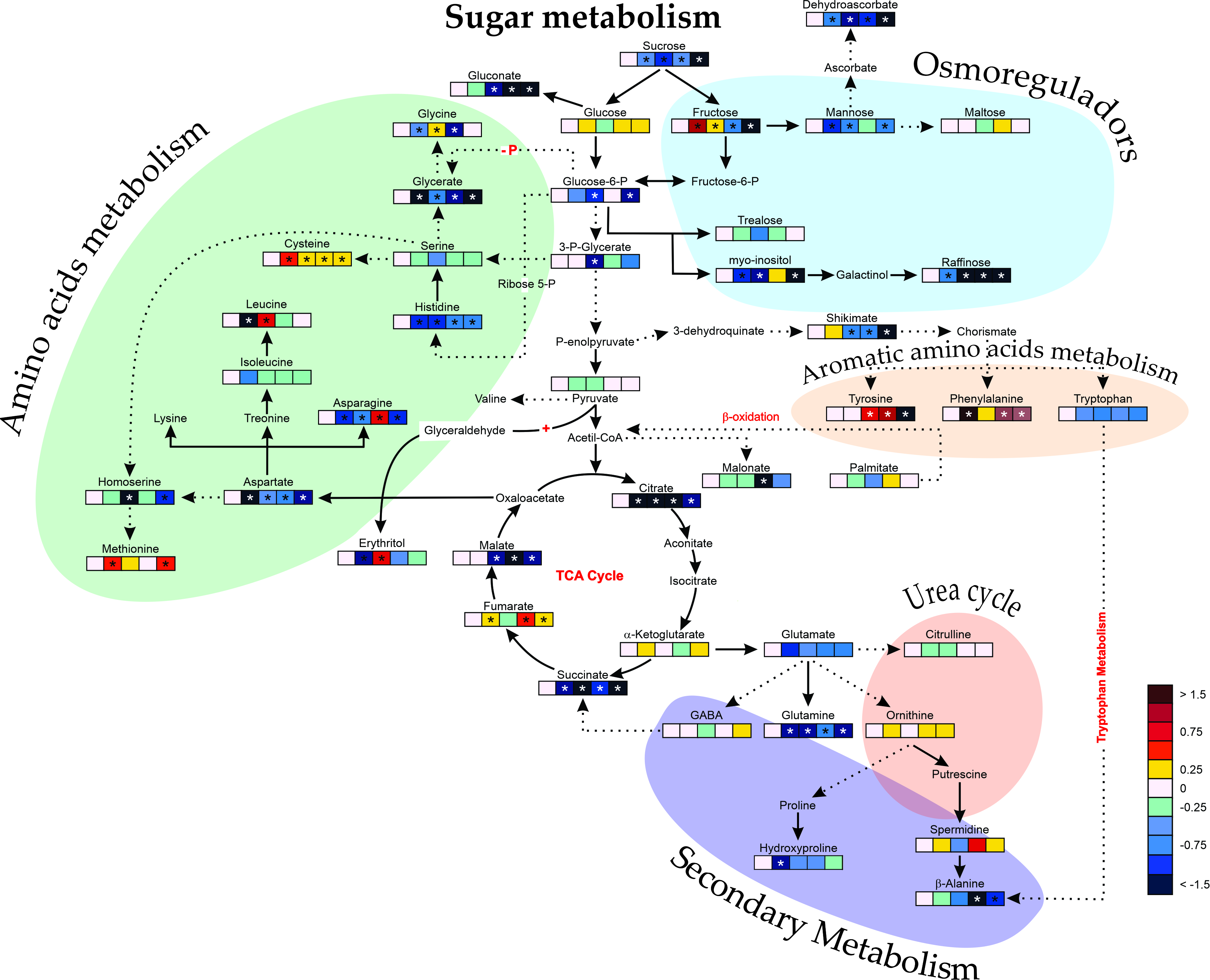

Supplement: Supplementary file 1 [file plants-13-01906-s001.zip › Supplementary Figure S1.tif]

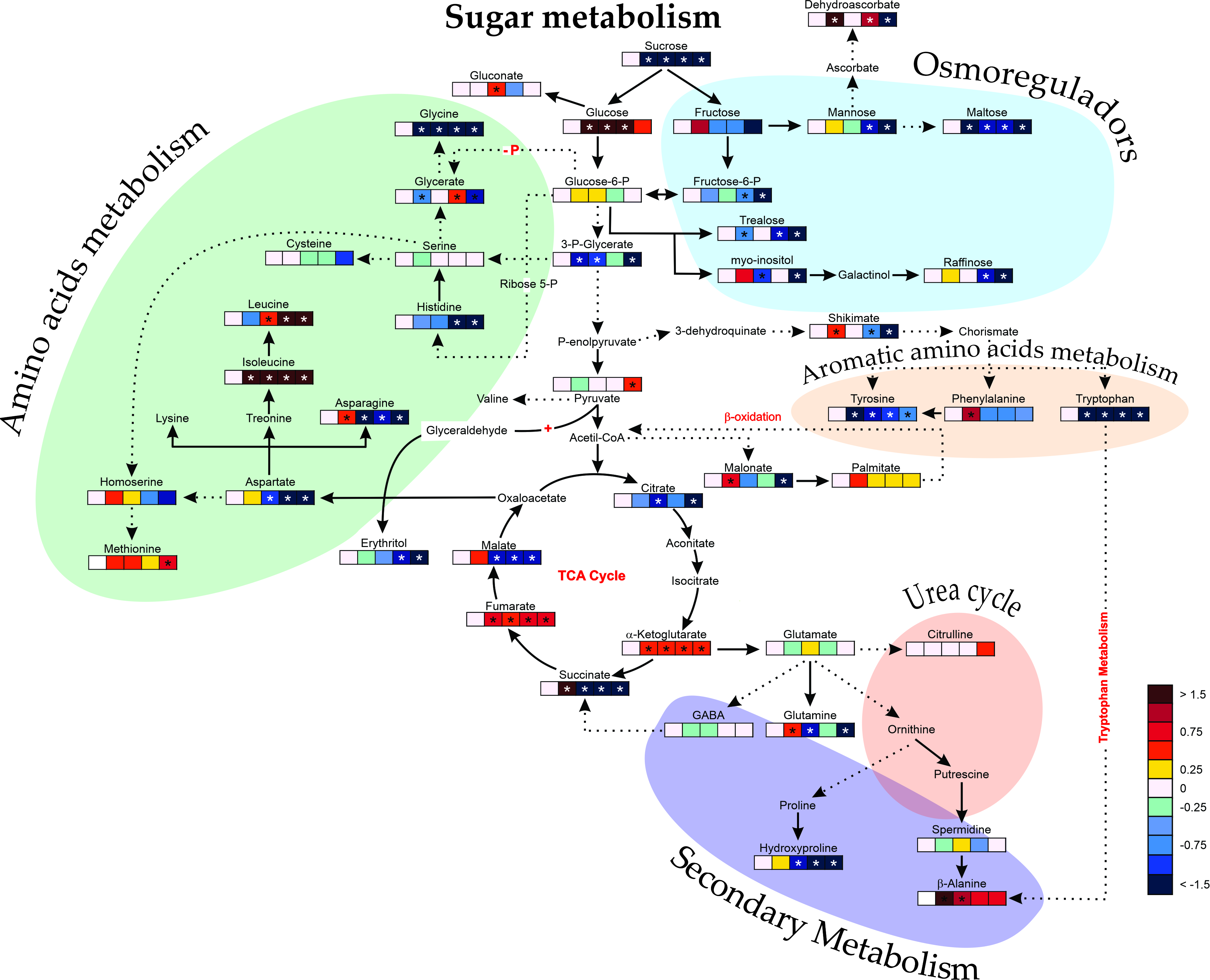

Supplement: Supplementary file 1 [file plants-13-01906-s001.zip › Supplementary Figure S2.tif]

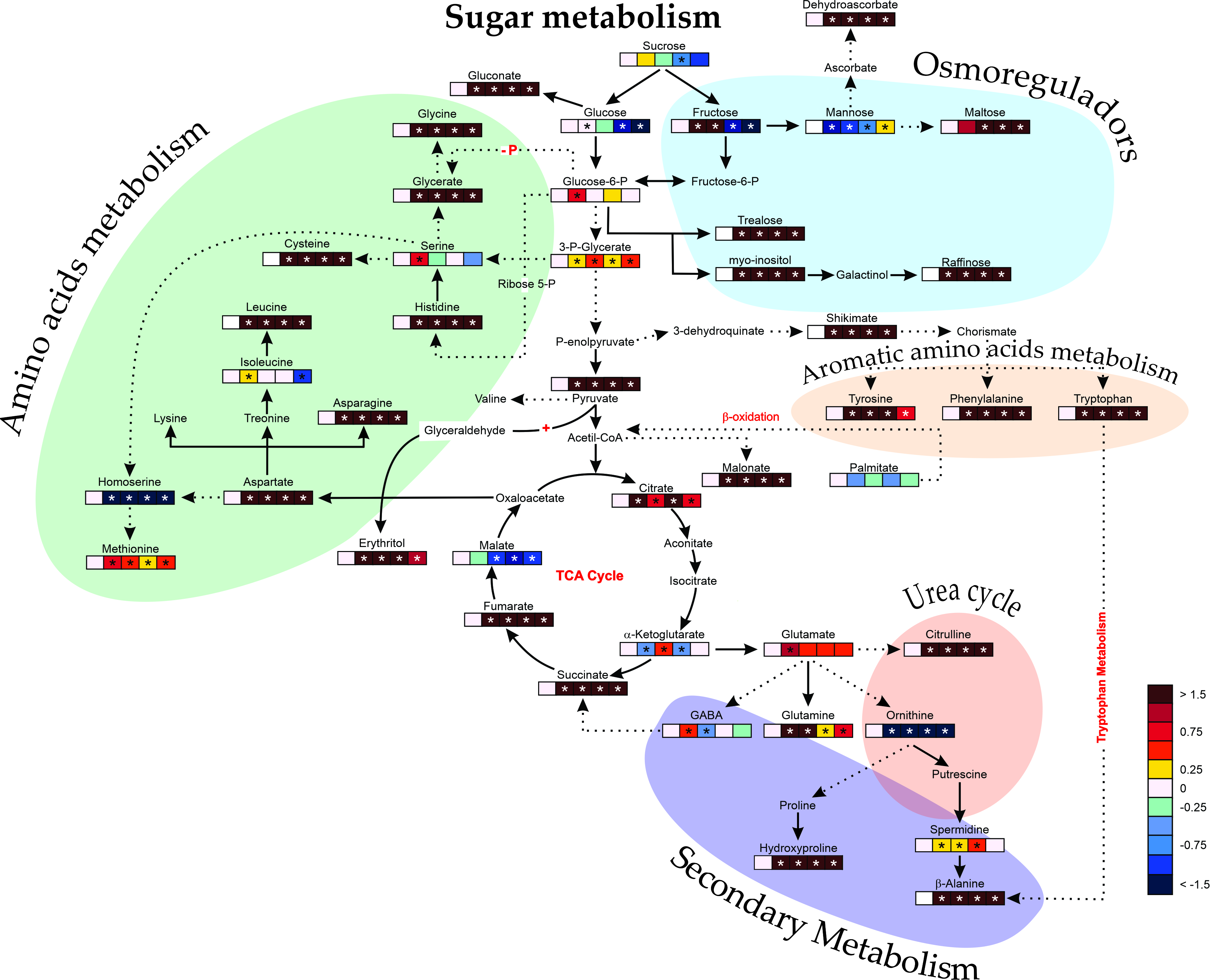

Supplement: Supplementary file 1 [file plants-13-01906-s001.zip › Supplementary Figure S3.tif]

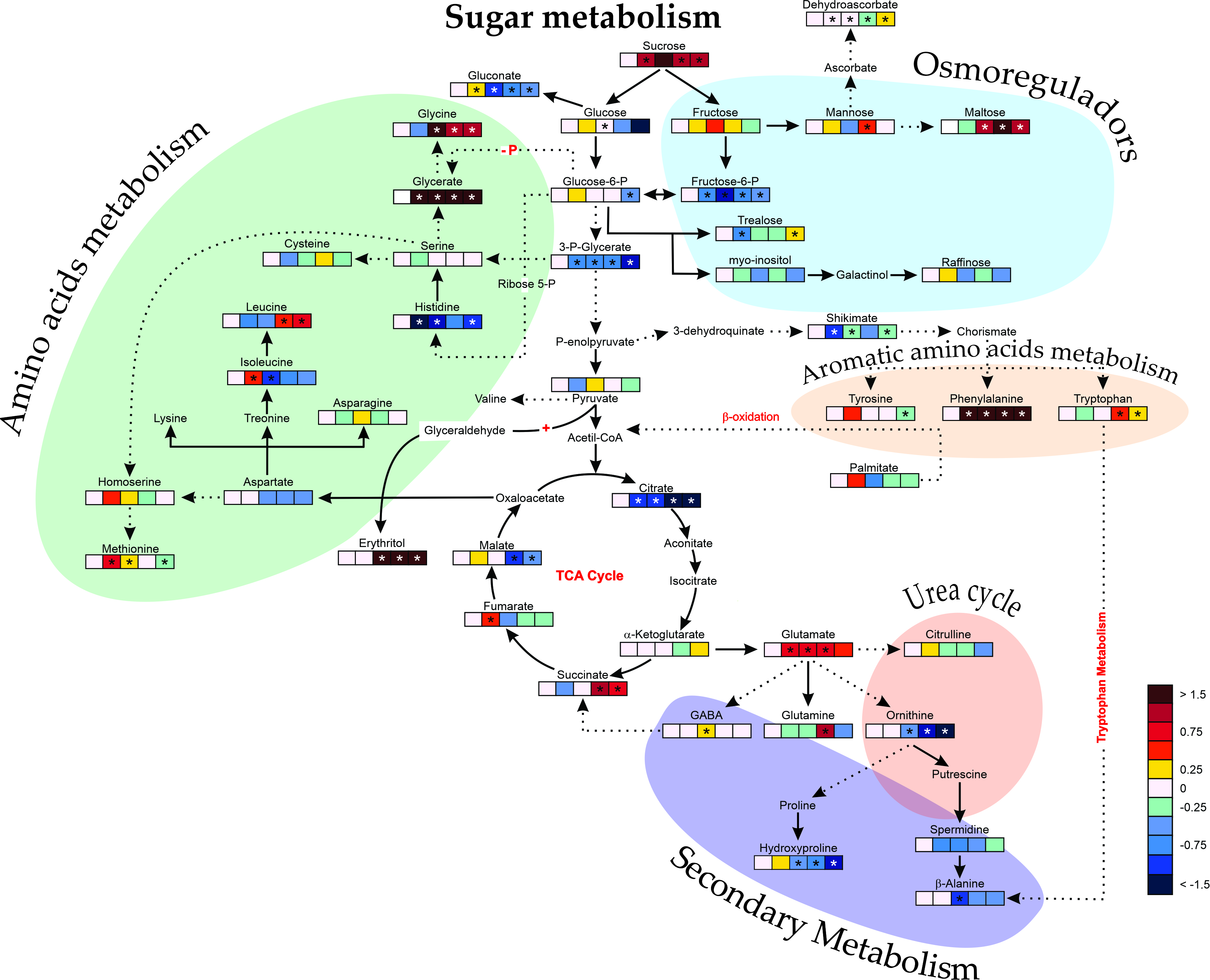

Supplement: Supplementary file 1 [file plants-13-01906-s001.zip › Supplementary Figure S4.tif]

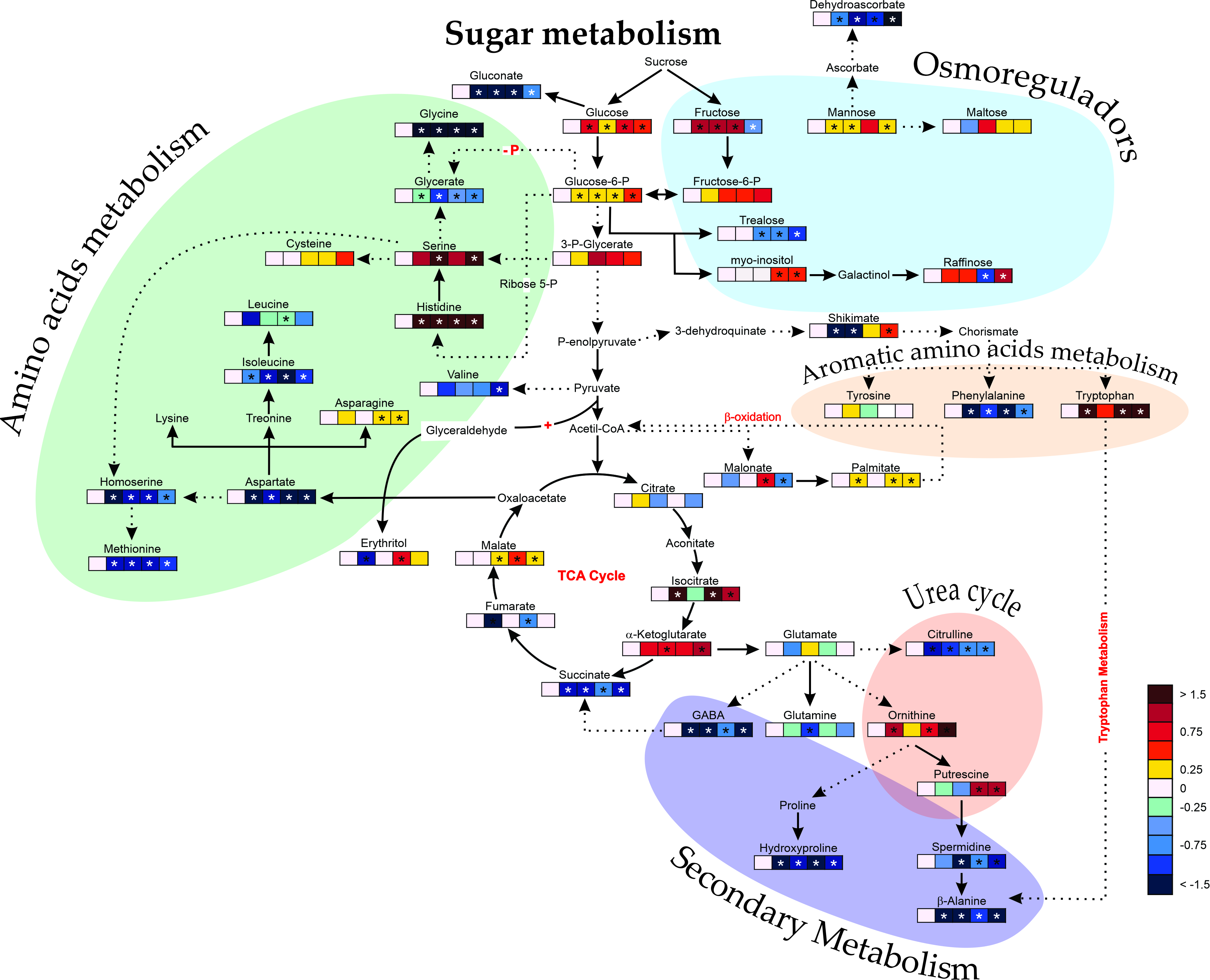

Supplement: Supplementary file 1 [file plants-13-01906-s001.zip › Supplementary Figure S5.tif]

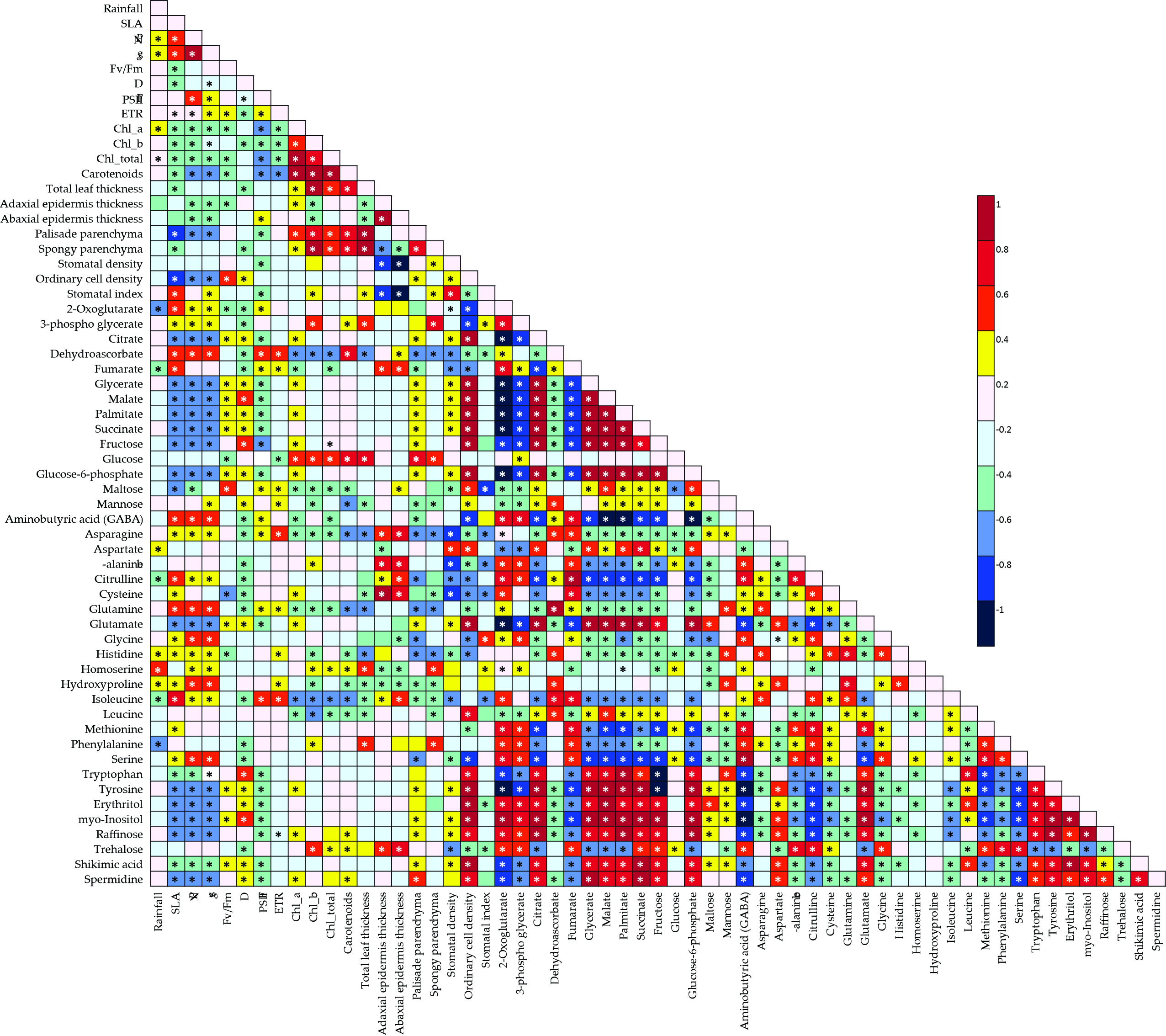

Supplement: Supplementary file 1 [file plants-13-01906-s001.zip › Supplementary Figure S6.tif]
